# Fish of the West

**Published:** 1853-07

**Authors:** 


*Fish of the West.*

THE distinguished naturalist, Louis Agassiz, passed through our city not long since on his return from a short tour through the South and West.

His object in visiting this section was to make such arrangements as would enable him to obtain a complete collection of fish from our western waters, preparatory to a classification and description of them in a new work upon this department of natural history, which he is now preparing.

Subscribers to this Journal, and others interested in the subject, would aid the cause of science and do a great favor to one of her most devoted votaries by sending such specimens of fish as may be found in the waters of their vicinity, to Prof. Agassiz, Cambridge, Mass., or to the editors of this Journal, who will transmit to him, with the name of the donor, all such contributions. They should be packed in alcohol, the large fish in casks, the small ones in jars.] H.

